# COVID-19 incidence and cardiorespiratory fitness among first-year college students

**DOI:** 10.3389/fpubh.2024.1468300

**Published:** 2024-10-15

**Authors:** Catherine E. Alvaro, Kyle S. Levers, Matthew D. Barberio, Yichen Jin, Andrew M. Stranieri, Jennifer M. Sacheck

**Affiliations:** Milken Institute School of Public Health, Department of Exercise and Nutrition Sciences, The George Washington University, Washington, DC, United States

**Keywords:** COVID-19, cardiorespiratory fitness, physical activity, college students, perceived health

## Abstract

**Objective:**

With the emergence of the COVID-19 virus, there was a widespread infection rate among college campuses, creating a need to understand the impact of COVID-19 infection on the health and wellbeing of adolescents. The aim of this study was to examine COVID-19 incidence and cardiorespiratory fitness (CRF) among undergraduate students in the 2 years post-COVID-19 pandemic lock-down.

**Participants:**

Participants (*n* = 151) included undergraduate college freshmen students during the 2021–2022 and 2022–2023 academic years.

**Methods:**

A series of annual surveys (*n* = 151) and laboratory measures among a sub-sample of participants (*n* = 28) were conducted to assess COVID-19 incidence, CRF, anthropometrics, and physical activity (PA).

**Results:**

Over half of participants self-reported COVID-19 infection (59%), “good” or better CRF, a healthy body mass index (BMI), and 25% met PA recommendations. Nearly a quarter, (24%) perceived a negative impact of COVID-19 on their CRF and although not statistically significant, participants who contracted COVID-19 had 46% lower odds of having a positive perception of CRF than those who did not contract COVID-19 before. However, students who were more physically active were more likely to perceive a negative impact of COVID-19 incidence on their CRF (*p* = 0.035).

**Conclusion:**

Although no relationship was detected between COVID-19 and CRF, those who perceived a negative impact of COVID-19 on their CRF reported engaging in more physical activity.

## Introduction

In 2020, the rapid spread of SARS-CoV-2 (COVID-19) created a global pandemic that resulted in quarantines and limited in-person contact. For undergraduate students in the United States (U.S.), this led to an unexpected cancelation of in-person classes or transition to remote, virtual learning to mitigate contraction of the highly contagious virus. Most universities returned to in-person learning, with precautions in place, during the 2021–2022 school year. Despite efforts to reduce the spread of the virus, the Centers for Disease Control and Prevention (CDC) reported over 100 million COVID-19 cases total in the U.S. at the end of 2022 (1) with college campuses serving as the grounds for super spreader incidences (2).

The coronavirus family is responsible for acute respiratory tract infections, with SARS-CoV and MERS-CoV viruses resulting in severe acute respiratory syndrome (SARS) that can lead to respiratory failure (3, 4). Common symptoms of COVID-19 present as fever, cough, fatigue, dyspnea, and sputum (5); though, the virus also impacts the cardiovascular system (6). Endothelial dysfunction, damage to the myocardium, and changes to the right and left ventricular structures result in reduced cardiovascular function leading to poor health outcomes especially in individuals with elevated cardiovascular risk factors (e.g., pre-existing cardiometabolic disease, auto-immune diseases, and cancer, etc.) (7). At least one coronary heart disease risk factor has been reported among more than one half of young adults aged 18–24 years (8). As a result, there is a need to understand the effects of acute COVID-19 infections on cardiovascular function and fitness across the college years, even in the absence of pre-existing risk factors. A review of available post-COVID-19 infection studies demonstrates a reduction in cardiorespiratory fitness (CRF) brought about by a sequelae of physiological changes, such as reduced lung diffusion of gasses, cardiac output and stroke volumes, in addition to peripheral changes in oxygen extraction and mitochondrial function (9). COVID-19 infections resulting in reduced cardiovascular function may lead to increased risk for cardiovascular disease (CVD), decreased quality of life, and increased risk of all-cause mortality throughout the lifespan. There is limited information about the impact of COVID-19 on CRF of young individuals despite evidence that COVID-19 may result in limitations in and damage to the cardiovascular system in this population.

Irrespective of the cardiorespiratory factors associated with COVID-19 infection, low CRF is associated with greater risk for all-cause mortality, CVD events, and cancer mortality in adults as well as associated with cardiometabolic risks in adolescents (10–12). A nationally representative sample in the U.S. demonstrated that only 42% of adolescents aged 12–15 years old had healthy CRF (13). Additionally, a decline in mean CRF of 0.9 mL/kg/min per decade has been reported between 1995 and 2013 in children in the U.S. aged 9–17 years (14). Physical activity is also a significant factor in the health status of children and adults, associated with cardiorespiratory fitness (15, 16). Low CRF can be prevented with lifestyle modifications, such as an increase in physical activity levels (17). In the context of college students transitioning from childhood into young adulthood, there is a reported decline in physical activity when enrolling in university (18, 19). Regular moderate-to vigorous physical activity and elevated CRF levels are promoted as preventative measures of CVD, especially with the concern of pervasive physical inactivity and sedentary behaviors worldwide (20). Since the start of the COVID-19 pandemic, patients with CVD increased sedentary time by 28% and decreased recreational physical activity by 33% (20). An early systematic review of university students of different countries reported a significant decline in physical activity levels, with a decrease in mild physical activity between 32.5 and 366% and a decrease in vigorous physical activity between 2.90 and 52.8%, during the COVID-19 pandemic when compared to pre-pandemic physical activity levels (21).

Therefore, the purpose of this study was to investigate among college freshman in the two academic years following the post-COVID lock-down: (1) differences in CRF (self-reported and measured), physical activity, anthropometrics by COVID-19 incidence; (2) associations between COVID-19 incidence and perceived CRF; and (3) to explore among those who perceived that COVID-19 incidence impacted their CRF, whether there were any differences in their measured CRF, physical activity, or anthropometrics.

## Methods

### Study design

The FRESH (Fitness, Rest, and Energy for Strength and Health) Study began in the fall of 2021 and is designed to annually examine the health behaviors and indicators of first year undergraduate college students at the George Washington University (GWU; a mid-size urban university in Washington, D.C.), and follow them over their four-year undergraduate experience using surveys and laboratory-based fitness assessments. Surveys include questions from validated instruments encompassing physical activity, diet, sleep, stress, and general health. For the present study, survey data collected on first-year undergraduate students from the 2021–2022 and 2022–2023 academic year cohorts along with laboratory fitness testing among both cohorts were utilized. The survey included questions on COVID-19 incidence and symptoms as well as perceived CRF and physical activity levels. The laboratory assessments provided additional in-person measurements including CRF, body composition, and height and weight for assessment of BMI. Survey and laboratory assessment data were collected and managed using REDCap electronic data capture software (22, 23). We examined the relationship between self-reported COVID-19 incidence and both perceived and measured CRF, as well as correlates of CRF (physical activity and anthropometrics). This study was approved by the George Washington University Institutional Review Board prior to participant recruitment.

### Recruitment

All first-year undergraduate students at GWU were eligible to participate in the FRESH Survey. Exclusion criteria for the laboratory fitness testing included injuries that would interfere with assessments and self-reported diagnosed medical conditions including, but not limited to, cardiovascular, pulmonary, and/or orthopedic pre-existing conditions. As of 2022, there were a total of 10,798 undergraduate students at GWU with about 45.8% of the population identifying as White, 12.3% as Asian, 10.2% as Hispanic, 10.1% as Black, and 62.3% as women (24). Recruitment procedures entailed distributing study information via email, group message, social media, and physically as a handout to reach the first-year undergraduate student population on campus. First-year undergraduate advisors, courses, organizations, dormitories, dining halls, and events as well as general high-traffic campus areas were specifically targeted. Participant informed consent was acquired before participant enrollment. A small stipend of $10 was provided to each participant for completing the survey and separately $25 for completing the laboratory assessment visit. The participants included were part of the pilot study, which occurred post COVID-19 pandemic during a period in which restrictions were imposed on in-person activities at the university. A total of 151 students were recruited, and 28 of them completed laboratory sub-study assessments.

### FRESH Study Survey

The FRESH Study Survey was administered through a link to REDCap (22, 23) shared by email and included the sections: demographics, general health, perceived stress scale, sleep quality index, physical activity, dietary intake, beverage consumption, and sports participation. The study surveys were released from February to mid-March of 2022 and the next freshmen group of surveys were released February of 2023 through an email with a link to REDCap (22, 23). Participants completed the surveys individually and remotely. Below are the components from the FRESH Study Survey considered in this study.

#### Demographics and anthropometrics

Demographic questions included date of birth, biological sex assigned at birth, gender identity, race, ethnicity, primary language spoken at home, and academic year. Self-reported height and weight were collected for calculation of BMI according to CDC guidelines and categorized BMI less than 18.5 as Underweight, BMI between 18.5 and 25.0 as normal weight, BMI between 25.0 and 29.9 as with overweight, and BMI greater than or equal to 30.0 as with obesity (25).

#### COVID-19 questionnaire

Questions included from the COVID Crisis Survey inquired about COVID-19 incidence, number of distinct infections, and the date of infection as well as how diagnosed (26). Additionally, questions were posed in relation to COVID-19 incidence, symptoms experienced, and perceived negative impacts to cardiorespiratory endeavors. Participants were asked to rate their current CRF levels and their CRF levels before and after contracting COVID-19, compared to their age group. Those who did not contract COVID-19 had the option N/A. Cardiorespiratory fitness was defined as heart and lung function to power physical activity, with the example of breathing rate while exercising. The CRF question responses offered were Very Poor (0–19 percentile), Poor (20–39 percentile), Fair (40–59 percentile), Good (60–79 percentile), Excellent (80–94 percentile), and Superior (95+ percentile). The COVID-19 questionnaire (Appendix I) was administered before the commencement of any fitness testing to each participant in the 2022–2023 cohort and only participants who completed fitness testing in the 2021–2022 cohort due to COVID-19 restrictions. The variable *General Perception of Fitness* characterized responses to the questions from the COVID-19 Questionnaire, “How would you rate your current overall CRF compared to your age group?.” *Positive* classification included Superior, Excellent, and Good responses. *Negative* classification included Fair, Poor, and Very Poor responses. The question “Do you think your cardiorespiratory endeavors or ability to engage in physical activities were negatively impacted in the weeks after your initial COVID-19 contraction?” was labeled as the variable *Yes impact* and *No impact* for statistical analyses.

#### Physical activity questionnaire

This survey was adapted to include a series of questions from the 2002 International Physical Activity Questionnaire (IPAQ) (27) about physical activity habits over the last 7 days, prior to taking the survey. Questions were grouped into categories of vigorous, moderate, walking, sitting, and electronic-based activities. Physical activity was calculated by combining the self-reported minutes of vigorous and moderate physical activity the participants engaged in the past week.

### Laboratory assessments

Survey participants were invited for a pilot sub-study which included fitness assessments in the Metabolic and Exercise Testing Laboratory at GWU. Prior to the laboratory testing session, an email was sent out to participants with pre-testing guidelines for the day before the test and the day of the test.

#### Anthropometric data

Height was measured and recorded as the average of three measurements in the laboratory by research assistants, and weight was recorded from the InBody multi-frequency Bioelectrical Impedance (BIA) test (InBody 770, Cerritos, CA, USA). The height and weight measurements were used to calculate BMI (25). Body composition information from dual-energy X-ray absorptiometry (DXA) was used to evaluate total percent body fat (% BF). DXA scans were conducted following standard testing techniques and preparation (28).

#### Cardiorespiratory fitness

CRF was determined using the Bruce submaximal aerobic capacity treadmill protocol using a chest strap heart rate monitor (Polar Electro, Kempele, Finland), a reliable and valid tool (29–31), to furnish stage-based heart rate (HR) alongside recording rating of perceived exertion (RPE) data (32–34). Laboratory outcomes were collected and input into REDCap (22, 23). Before participating in the submaximal Bruce protocol (32–34), each participant was led through a standardized warm-up to raise, activate, mobilize, and potentiate the neuromuscular and cardiorespiratory systems. Each participant was given a Polar H10 heart rate monitor and chest strap (Polar Electro, Kempele, Finland) to wear at the level of the sternum for the duration of the CRF protocol. A standard Borg 6–20 RPE scale (35) was also collected alongside HR at rest, at the end of each Bruce protocol 3-min stage, and every minute throughout the post-test recovery period. As stated by Fletcher et al. (33), 85% of the participant’s age predicted maximum heart rate (APMHR) was used as the primary stop test criterion. The Bruce submaximal aerobic capacity treadmill test was conducted on a belt-driven, self-calibrating Trackmaster motorized treadmill (Full Vision Inc., Newton, KS, USA), following established procedures as previously described (32, 34). Estimated VO_2_max was calculated using submaximal test completion time in minutes within the following equation: VO_2_max (mL/kg/min) = 14.8 – (1.379 ∗ time) + (0.451 ∗ time^2^) – (0.012 ∗ time^3^) (32).

### Analyses

All analyses were performed using R (version 4.3.3). Sociodemographic characteristics and anthropometrics were presented as *n*, percentage, mean (standard deviation), or median (interquartile range). Outliers (>3 standard deviations) of physical activity (*n* = 3) and BMI (*n* = 1) were treated as missing values and excluded from the survey analysis of physical activity and weight status, respectively.

Differences in CRF, physical activity, and anthropometrics by COVID-incidence (yes/no) was examined among the survey respondents (*n* = 151) as well as the sub-sample that participated in the laboratory assessment visit (*n* = 28) using Student’s *t*-test for continuous variables and chi-square test for categorical variables. Using linear regression analyses, associations were separately examined between: (1) self-reported fitness (survey respondents) and perceived impact of COVID-19 infection; and (2) CRF (lab participants) and perceived impact of COVID-19 infection incidence (VO_2_max was adjusted for sex in the model). A logistic regression model was used to examine the association between CRF and COVID-19 incidence for survey data adjusting for weight status. Normality of the continuous variables was tested, and the Mann–Whitney U test was used to compare the medians between groups with variables that did not pass the normality test. Statistical significance was set to *p* < 0.05.

## Results

First year undergraduate students (*n* = 151; 80% female; 18.9 ± 0.4 yrs.; 23.4 kg/m^2^ female vs. 23.0 kg/m^2^ male) from the 2021–2022 (*n* = 71) and 2022–2023 (*n* = 80) cohorts were included in this study and completed the FRESH Survey (Table 1). Twenty-eight (*n* = 28) of these students (18.9 ± 0.4 yrs.; 25.3 kg/m^2^ female vs. 24.1 kg/m^2^ male) also participated in the laboratory-based sub-study to measure CRF and anthropometrics. (In-person laboratory data was conducted post COVID-19 pandemic when university facilities reopened under restricted activity guidelines, which limited student participation in the laboratory portion of the study.) The study included 58.3% of participants identifying as Caucasian/White, 17.9% as Asian, 9.3% as Hispanic/Latino, 15.2% as African American/Black, and 5.3% as multiracial.

**Table 1:** Descriptive statistics and self-reported COVID-19 incidence of freshman undergraduate students participating in the FRESH Study.

	2021–2022 Cohort	2022–2023 Cohort	Total (*n*, %)
Participants (*n*, %)	71, 47%	80, 53%	151, 100%
Biological sex at birth (*n*, %)			
Male	11, 16%	19, 24%	30, 20%
Female	60, 85%	61, 76%	121, 80%
Race/ethnicity (*n*, %)			
African American/Black	7, 10%	16, 20%	23, 15%
Caucasian/White	43, 61%	45, 56%	88, 58%
Asian	13, 18%	14, 18%	27, 18%
Hispanic/Latino	7, 10%	7, 9%	14, 9%
Other	4, 6%	1, 1%	5, 3%
Multiracial	4, 6%	4, 5%	8, 5%
COVID-19 incidence (*n*, %)			
Yes	40, 56%	49, 61%	89, 59%
No	31, 44%	31, 39%	62, 41%

### COVID-19 incidence

At the time of survey administration, 58.9% (*n* = 89) of first-year undergraduate students reported they had contracted COVID-19. Of those who had COVID-19, 58.4% (*n* = 52) were symptomatic and 51.9% (*n* = 27) had symptoms lasting more than 3 days with the remaining cohort (48.1%, *n* = 25) reporting symptoms for 3 days or less. The most reported symptoms among those who contracted COVID-19 and were symptomatic included cough (79%, *n* = 41), headache (69%, *n* = 36), aches (62%, *n* = 32), and sore throat (62%, *n* = 32).

### Anthropometrics and physical activity

Calculated BMI (23.5 ± 4.5 kg/m^2^) via self-reported height and weight data indicate that 25% of participants (26% female and 23% male) were with overweight or with obesity. Self-reported daily participation in moderate to vigorous physical activity averaged 344.4 min/week [49.2 min/d, 24.7% met weekly physical activity guidelines of 150 min/week (34)] with a median of 240 min/week [7.5, 480] for participants who completed the survey.

### Perceived health and cardiorespiratory fitness

Prior to COVID-19 infection, participants indicated that their CRF was superior (17%, *n* = 13), excellent (28%, *n* = 22), good (37%, *n* = 29), fair (17%, *n* = 13), or poor (1%, *n* = 1). However, following initial COVID-19 infection, almost one quarter (23.6%) of the participants reported that COVID-19 negatively impacted their CRF and their ability to engage in physical activity and exercise. Among participants who filled out the open response, 69% reported cardiorespiratory endeavors felt more strenuous, 19% reported increased heart rate, 44% reported difficulty breathing, and 13% reported exacerbated asthma symptoms in the months following COVID-19 infection.

There were no significant differences in self-reported CRF, weekly physical activity engagement, or weight status between participants who reported COVID-19 infection and those who did not (Table 2). Similarly, there was no significant association between COVID-19 incidence and perceived fitness after adjustment for weight status (odds ratio 0.56 [95% CI: 0.14–1.92], *p* = 0.378). Although not significant, participants infected with COVID-19 had 46% lower odds of having a positive perception of fitness compared to those who did not previously contract COVID-19.

**Table 2:** Self-reported health measures stratified by COVID-19 incidence among FRESH Study Survey participants (*n* = 151).

	Yes COVID-19 (*n* = 89)	No COVID-19 (*n* = 62)	*p*-value
Fitness level (positive *n* = 96, %)	46, 82%	36, 90%	0.43^a^
Weight status (BMI, *n* = 150, %)			0.97^a^
Underweight	4, 5%	3, 5%	
Normal weight	61, 69%	44, 71%	
Overweight	17, 19%	12, 19%	
Obese	6, 7%	3, 5%	
Physical activity (*n* = 148, min/week, median [IQR])	265 [0, 457.5]	165 [17.5, 480]	0.48^b^

BMI = body mass index (kg/m^2^) from self-reported height and weight.

Physical activity = self-reported minutes of moderate-to-vigorous activity per week.

Fitness = self-reported cardiorespiratory fitness as “good,” “excellent,” or “superior”.

a Chi-square test for categorical variables.

b Mann-Whitney U test for continuous variables with non-normal distribution.

### Laboratory exploratory sub-study

Among the sub-sample that participated in laboratory assessments (*n* = 28; Table 3), the average estimated maximal aerobic capacity (VO_2_max) was 25.0 mL/kg/min (median of 23.13 mL/kg/min [20.93, 26.50]) among females and 29.3 mL/kg/min (median of 28.35 mL/kg/min [23.97, 34.11]) among males, both categorized in the very poor range (32). They self-reported ~60 min per day of physical activity (42.9% met weekly PA guidelines) [201, 585]. Measured BMI and body composition were 24.9 ± 5.3 kg/m^2^, and 28.6 ± 9.9%, respectively with approximately one third with overweight or with obesity.

**Table 3:** Health and cardiovascular fitness measures stratified by COVID-19 incidence among FRESH Study participants who completed laboratory fitness testing (*n* = 28).

	Yes COVID-19 (*n* = 14)	No COVID-19 (*n* = 14)	*p*-value
VO_2_max (mL/kg/min, median [IQR])	24.8 [23.1, 33.1]	26.5 [23.1, 26.5]	0.87^b^
RPE [mean (SD)]	14.3 (2.6)	13.4 (3.0)	0.39^c^
Fitness level (perceived positive, *n*, %)	11, 79%	13, 93%	0.59^a^
Weight status (BMI, *n*, %)			0.39^a^
Underweight	0, 0%	1, 7%	
Normal weight	8, 57%	10, 71%	
Overweight	2, 14%	2, 14%	
Obese	4, 29%	1, 7%	
Body fat [mean (SD)]	29.3 (10.5)	27.9 (9.5)	0.70^c^
Physical activity (min/week, median [IQR])	360 [300, 885]	285 [120.75, 450]	0.13^b^

BMI = body mass index (kg/m^2^) from measured height and weight.

Fitness = self-reported cardiorespiratory fitness as “good,” “excellent,” or “superior”.

RPE = VO_2_max test rating of perceived exertion.

VO_2_max *p*-value adjusted for sex.

a Chi-square test for categorical variables.

b Mann-Whitney U test for continuous variables with non-normal distribution.

c Independent sample *t*-test for continuous variables with normal distribution.

There were no significant differences in CRF, ratings of perceived exertion during the CRF test, or body composition measures between those who reported COVID-19 infection and those who did not. Only three out of the 14 participants (21.4%) that reported COVID-19 infection perceived that it negatively impacted their CRF (Table 4). These three participants reported engaging in more than twice the weekly moderate-to-vigorous physical activity compared to the other laboratory-tested participants reporting COVID-19 infection (960 ± 183 vs. 330 ± 326 min/week, *p* = 0.035), however there were no significant differences in measured CRF or body composition/weight status.

**Table 4:** Reported and measured values of health and cardiovascular fitness by perceived impact of COVID-19 incidence on fitness among FRESH Study participants (lab sub-sample; *n* = 14 reported COVID-19 incidence).

	Yes impact (*n* = 3)	No impact (*n* = 11)	*p*-value
VO_2_max (mL/kg/min, median [IQR])	34.1 [28.6, 36.2]	23.1 [21.7, 28.4]	0.27^b^
RPE [mean (SD)]	12.0 (1.0)	14.9 (2.6)	0.091^c^
Fitness level (perceived positive, *n*, %)	3, 100%	8, 73%	0.82^a^
Weight status (BMI, *n*, %)			0.73^a^
Underweight	0, 0%	0, 0%	
Normal weight	2, 67%	6, 55%	
Overweight	0, 0%	2, 18%	
Obese	1, 33%	3, 27%	
Body fat [mean (SD)]	20.7 (15.5)	31.7 (8.1)	0.11^c^
Physical activity (min/week, median [IQR])	960 [900, 1,080]	330 [227.5, 395]	0.035^b^*

BMI = body mass index (kg/m^2^) from measured height and weight.

Fitness = self-reported cardiorespiratory fitness as “good,” “excellent,” or “superior”.

RPE = VO_2_max test rating of perceived exertion.

VO_2_max p-value adjusted for sex.

a Chi-square test for categorical variables.

b Mann-Whitney U test for continuous variables with non-normal distribution.

c Independent sample *t*-test for continuous variables with normal distribution.

*p < 0.05.

## Discussion

Our findings demonstrate that the majority of college students with a history of COVID-19 infection generally did not have any differences in perceived CRF, PA engagement, or weight status. Although not statistically significant, participants who had COVID-19 had lower odds of having a positive perception of their CRF compared to those never infected. Furthermore, although we hypothesized that contracting COVID-19 may have a more perceptible impact on CRF of those who are less fit and less physically active college students, students who perceive a negative impact of COVID-19 infection on their CRF are actually more physically active. Thus, detecting any negative impact of COVID-19 incidence on optimal post-infection physical activity engagement is likely affected by the pre-infection physical activity baseline and CRF perception. Those who engaged in greater physical activity are more likely to notice a change in their CRF and associated physical activity limitations post-COVID-19 contraction than those who are not as physically active and less perceptive about their aerobic fitness.

The Physical Activity Guidelines for Americans recommends that healthy adults ages 18–65 years old engage in moderate intensity physical activity for at least 30 min five days per week or vigorous intensity physical activity for at least 20 min three days per week (36). In this study, 58% of participants reported exceeding the minimum recommended weekly levels of physical activity, however, the majority of participants who completed voluntary laboratory testing were categorized as very poor to poor based on their CRF and aerobic capacity results. The discrepancy between high levels of physical activity engagement and low values of estimated maximal aerobic capacity may be attributed to participants miscalculating or overestimating physical activity engagement (37). Low levels of estimated maximal aerobic capacity may be due to participants attaining a VO_2_peak rather than a VO_2_max (38). Though 85% of their age-predicted maximum heart rate was achieved, the testing protocol may have captured a symptom-limited peak rather than an upper functional limit due to the limited exposure to higher intensity physical activity in this population.

The reported physical activity engagement of participants in the current study is similar to Spring 2022 data reported by the American College Health Association (ACHA), where approximately 69% of undergraduate students reported meeting the Physical Activity Guidelines for aerobic activity (39). However, the ACHA survey does not include measures or perceptions of CRF, nor does it provide stratified data by year of college enrollment (39). A discrepancy between college students’ self-reported physical activity and accelerometer-measured physical activity has been reported, with self-reported data classifying 66.7% of students as sufficiently physically active compared to accelerometer-measured data classifying only 33.8% of students as sufficiently physically active (37). Therefore regardless of reported physical activity levels, it is likely that students over report their activity levels and attention to the continued promotion of physical activity in this population is warranted. It is further noteworthy that a cohort study of adult patients at Kaiser Permanente with a positive COVID-19 diagnosis reported a higher incidence of hospitalization and poor COVID-19 outcomes among patients classified in lower physical activity engagement categories (40). Therefore, pre-infection physical activity participation may be a significant determinant in the severity of COVID-19 outcomes. Additionally, our study included a predominance of female participants (80%), potentially reducing the generalizability of our findings across genders, though the GWU student body in 2022 was predominantly women (62.3%) (24). Data collection for this study is ongoing for future research to draw stronger causal inferences about the impact of COVID-19 on CRF in a longitudinal manner.

At the time of this study, there have been over 700,000 COVID-19 cases among undergraduate students in the U.S. since the start of the pandemic (41). Data before the pandemic also indicates a high prevalence of CVD risk factors among college students, which may exacerbate the impact of COVID-19 infection in this population (8). Cardiorespiratory exercise testing research reveals that COVID-19 infection impacts the cardiorespiratory system through limitations in exercise tolerance post-contraction (42), specifically referencing depressions in cardiopulmonary responses to maximal exercise and reduced carbon monoxide diffusing capacity of the lungs (43). A decline in cardiorespiratory health due to COVID-19 infection alongside lower physical activity levels and reduced CRF, may significantly increase the risk of CVD development and incidence of cardiopulmonary-related events in younger populations (44). Symptoms of COVID-19 are exacerbated by CVD risk factors, including hypertension, diabetes, weight, and physical inactivity (45). In our research, we found that students who did not perceive a post-viral negative impact on their CRF tended to exercise less frequently and, though not statistically significant, had higher % BF via body composition measurement. The presentation of CVD risk factors that have been reported to exacerbate COVID-19 symptoms raises concern in the college student population, particularly in instances when CRF effects are not perceived. COVID-19 infection will likely increase the risk of developing CVD in the lower physical activity engagement group due to greater difficulty increasing physical activity levels post-infection. Additionally, students who did not previously have high CVD risk but increased risk factor development as a result of the COVID-19 pandemic will also expedite the likelihood of CVD development compared to others in their age group.

The impact of COVID-19 infection may be perceptually less in those who are physically inactive and who deem themselves as having lower CRF due to their lack of exposure to more challenging activities and, as a result, a limited understanding of their general CRF limitations. Concerningly, the lower CRF impact perception of COVID-19 infection in this population may have a greater and longer lasting impact on their cardiorespiratory health in the absence of recognizable symptoms or significant changes in daily life functional capacities. Post-COVID-19, a cross-sectional study of U.S. adults reported reduced physical activity engagement among previously active participants compared to no change in physical activity engagement among previously inactive participants (46). The reported associations may coincide with a perception or lack of perception of negative impacts due to COVID-19. In the context of our population throughout the COVID-19 pandemic, students likely experienced a decrease in opportunities to engage in physical activity, with campuses and recreational facilities closing, and an increase in screen time, with learning moved to a virtual format.

To our knowledge, there is limited information examining COVID-19’s impact on CRF and associated health markers (physical activity and weight status/adiposity) among college students. Other studies have examined relationships between general physical activity and fitness levels post-COVID-19, yet there is still a deficiency of information surrounding COVID-19 incidence and impact on CRF (21, 47, 48). While we utilized survey questions from several validated instruments, there are inherent limitations to self-reported data including COVID-19 incidence and health metrics, which may be potentially skewed due to participants overreporting or underreporting health markers, such as height, weight, and physical activity engagement. Our results could be strengthened by improving representation of the student body and as well as increasing the sample size of participants that participated in laboratory fitness testing. Unfortunately, laboratory testing opportunities were initially more limited due to COVID-19 restrictions, such as mask mandates and restricted in-person activity on campus, as we worked through and emerged from the pandemic. While the sample size of laboratory assessment participants was small, it is a noted strength that we collected both objective and subjective measures of CRF and weight status during the pandemic. These measures together allowed us to formulate a more comprehensive picture of how COVID-19 infection may affect CRF among college students.

Further research regarding the impact of COVID-19 incidence on college student CRF with low levels of physical activity and aerobic fitness is necessary, particularly when their perceived CRF is self-reported as healthy. Following COVID-19 infection, college students require the proper resources and knowledge to make informed decisions about optimal and safe strategies to engage or re-engage in exercise and physical activity. This is specifically vital for inactive or sedentary populations with limited exercise frequency as COVID-19 symptoms may act as another barrier, exacerbating the chronic cardiorespiratory health implications (44). Additionally, it is necessary to develop testing and programming strategies that facilitate an optimal return to physical activity and exercise for the overall population. Our research demonstrates that even participants who were physically active with above average CRF perceived a challenge when returning to physical activity post-COVID-19 infection. Our research provides data post-COVID-19 infection detailing the health experiences of first-year college students to pinpoint areas that need to be addressed with future research.

## Conclusion

Our findings demonstrate that those who perceived greater impacts of COVID-19 incidence on CRF were more physically active. However, the lack of perceived COVID-19 infection impact on CRF by less active participants does not indicate that this population was unaffected. Though not statistically significant, participants who did not perceive an impact of COVID-19 on their CRF tended to have higher percent body fat and lower aerobic capacity compared to those who perceived a greater CRF impact. Further research examining the effects of COVID-19 incidence on perceived versus measured CRF among college students should be facilitated in larger cohorts. In this manner, the impact of COVID-19 symptoms on cardiorespiratory health must be better understood to assist with cardiovascular risk factor mitigation in college-aged populations. Additionally, information from the current study may inform public health strategies concerning post-COVID-19 infection return to activity protocols for college students to reduce long-term implications on cardiorespiratory health and provide methods to optimize return-to-activity timelines. The decision to re-engage in physical activity should be encouraged to reduce the severity of COVID-19 infection and delay the onset of CVD risk factors. In doing so, we can mitigate additional risk factors for chronic diseases, such as CVD, commonly linked to COVID-19 infection in young adults.

## Data Availability

The raw data supporting the conclusions of this article will be made available by the authors, without undue reservation.

## Appendix I COVID-19 questionnaire

For the following questions *“cardiorespiratory fitness”* is defined as heart and lung function to power physical activity (e.g., breathing rate while exercising).

1. How would you rate your current overall cardiorespiratory fitness compared to your age group?
  1. Superior (95+ percentile)
  2. Excellent (80–94 percentile)
  3. Good (60–79 percentile)
  4. Fair (40–59 percentile)
  5. Poor (20–39 percentile)
  6. Very Poor (0–19 percentile)
2. How would you rate your overall cardiorespiratory fitness compared to your age group pre-COVID-19?
  1. Superior (95+ percentile)
  2. Excellent (80–94 percentile)
  3. Good (60–79 percentile)
  4. Fair (40–59 percentile)
  5. Poor (20–39 percentile)
  6. Very Poor (0–19 percentile)
3. How would you rate your overall cardiorespiratory fitness compared to your age group post-COVID-19?
  1. Superior (95+ percentile)
  2. Excellent (80–94 percentile)
  3. Good (60–79 percentile)
  4. Fair (40–59 percentile)
  5. Poor (20–39 percentile)
  6. Very Poor (0–19 percentile)
4. When you contracted COVID-19, were you ever symptomatic?
  1. Yes
  2. No
  3. N/A
5. If Yes, about how long did you experience symptoms?
  1. 3 days or less
  2. More than 3 days
6. If Yes, what symptoms did you experience? (Check all that apply).
  1. Fever
  2. Bronchial congestion
  3. Cough
  4. Headache
  5. Lethargy
  6. Sore throat
  7. Aches
7. Do you think COVID-19 negatively impacted your cardiorespiratory endeavors or ability to engage in physical activities?
  1. Yes
  2. No
8. If Yes, please explain: ____________________
